# Multitranscriptome analyses of keloid fibroblasts reveal the role of the HIF-1α/HOXC6/ERK axis in keloid development

**DOI:** 10.1093/burnst/tkac013

**Published:** 2022-05-09

**Authors:** Qi Wang, Yixiu Zhong, Zhijia Li, Dingheng Zhu, Hongyan Lu, Pingjiao Chen, Changxing Li, Xuebiao Peng, Qian Li, Kang Zeng

**Affiliations:** Department of Dermatology, Nanfang Hospital, Southern Medical University, Guangzhou 510515, China; Department of Dermatology, Nanfang Hospital, Southern Medical University, Guangzhou 510515, China; Molecular Diagnosis and Treatment Center for Infectious Diseases, Dermatology Hospital, Southern Medical University, Guangzhou 510091, China; Department of Dermatologic Surgery, Dermatology Hospital, Southern Medical University, Guangzhou 510091, China; Department of Dermatology, Nanfang Hospital, Southern Medical University, Guangzhou 510515, China; Department of Dermatology, Nanfang Hospital, Southern Medical University, Guangzhou 510515, China; Department of Dermatology, Nanfang Hospital, Southern Medical University, Guangzhou 510515, China; Department of Dermatology, Nanfang Hospital, Southern Medical University, Guangzhou 510515, China; Department of Dermatology, Nanfang Hospital, Southern Medical University, Guangzhou 510515, China; Department of Dermatology, Nanfang Hospital, Southern Medical University, Guangzhou 510515, China

**Keywords:** Keloid, Fibroblast, HOXC6, HIF-1α, ERK, Transcriptome

## Abstract

**Background:**

A keloid is a disease of excessive fibrosis that is characterized by the aberrant proliferation of fibroblasts. However, the molecular mechanisms of fibroblasts during the development of keloids remain unclear. This study aims to identify new molecular targets that promote the proliferation and migration of keloid fibroblasts, providing new ideas for the prevention and treatment of keloids.

**Methods:**

We utilized bioinformatics tools to analyze data from keloid fibroblasts (KFs) available in the Gene Expression Omnibus (GEO) database to identify the key genes involved in keloid development. Homeobox C6 (*HOXC6*) emerged as a hub gene in KFs from the GEO database was verified in keloid tissue samples and KFs using reverse transcription-quantitative polymerase chain reaction, western blot (WB) and immunohistochemistry. Subsequently, the effects of downregulated *HOXC6* expression on the cellular behaviors of KFs were examined by performing Cell Counting Kit-8, flow cytometry, transwell migration and WB assays. Meanwhile, we performed transcriptome sequencing and gene set enrichment analysis (GSEA) to further explore HOXC6-related mechanisms and validated the signaling pathways by performing a series of experiments.

**Results:**

*HOXC6* was the top-ranking hub gene of KFs in microarray datasets from GEO and was upregulated in keloid tissue samples and KFs. Downregulation of *HOXC6* inhibited proliferation, migration and extracellular matrix (ECM) accumulation and promoted KF apoptosis. GSEA predicted that the hypoxia signaling pathway was associated with *HOXC6* in KFs. Transcriptome sequencing suggested that the extracellular regulated protein kinase (ERK) pathway was one of the downstream pathways of *HOXC6* in KFs. Our experiments confirmed that hypoxia-inducible factor-1α (HIF-1α) upregulates *HOXC6*, contributing to KFs proliferation, migration, apoptosis inhibition and collagen accumulation through the ERK signaling pathway.

**Conclusions:**

Our findings first revealed that *HOXC6* acts as an oncogenic driver in the molecular mechanisms of fibroblasts in keloids. The HIF-1α/HOXC6/ERK axis promotes proliferation, migration and ECM production by KFs, contributing to the progression of keloids. Taken together, HOXC6 may serve as a promising novel therapeutic target and new focus for research designed to understand the pathogenesis of keloids.

Highlights

- This study first revealed HOXC6 upregulation in keloid fibroblasts.
- This study first uncovered that HIF-1α positively regulates the expression of HOXC6 in keloid fibroblasts.
- This study demonstrated the important function of the HIF-1α/HOXC6/ERK axis in keloid fibroblasts in promoting the growth and invasion of keloids.

## Background

Keloid is a destructive disease that clinically manifests as persistent and uncontrolled growth of tissue beyond the original wound margin to the neighboring skin, causing severe pain in patients. Conventional keloid therapies, including surgery, laser therapy and intralesional steroid injection, may aggravate the condition and increase the risks of recurrence and malignancy [1,2]. Emerging studies have confirmed the crucial role of keloid fibroblasts (KFs) in keloids. Consistent with the ‘tumor-like’ nature of keloids, the number of KFs increased in keloid tissues, and they exhibited the characteristics of enhanced proliferation, migration and extracellular matrix (ECM) deposition and inhibited apoptosis [3]. Although some studies have shown that the transforming growth factor-β1 (TGF-β1), vascular endothelial growth factor, connective tissue growth factor and interleukin (IL)-6 and IL-8 pathways are involved in the cellular behaviors of KFs [1], no effective therapeutic target for keloid treatment has been discovered. Therefore, studies aiming to decipher the molecular mechanism of keloids are urgently needed.

Recent investigations have highlighted the efficiency of genetic studies and bioinformatics analyses as useful tools to explore the molecular mechanisms and to identify potential therapeutic targets of human skin diseases. Bioinformatics analyses have clarified molecular mechanisms and indicated hyperactivation of the IL-1/IL-36 axis, aiding in the treatment of pustular psoriasis [4]. In fibrotic skin disorders, RNA-sequence analysis revealed the heterogeneity of fibroblasts and elevated numbers of mesenchymal fibroblasts [5]. Therefore, we applied a series of bioinformatics tools to analyze the gene expression matrix of KFs and discover potentially novel biomolecules that contribute to keloid.

Homeobox (HOX) genes are a crucial family of genes related to developmental biology and include 39 different genes divided into four parallel chromosomal groups: *HOXA*, *HOXB*, *HOXC* and *HOXD* [6]. Based on accumulating evidence, *HOX* genes are involved in the regulation of skin development [7–9]. In developing skin, *HOXC4* is expressed at high levels, while *HOXA5*, *HOXA7* and *HOXB7* are expressed at lower levels, and *HOXB4* is nearly undetectable [7]. In addition, studies have suggested that the high expression of *HOX* genes promotes unregulated proliferation, while the low expression of *HOX* genes promotes apoptosis in the skin and other systems [7,10–12]. Therefore, some scholars have speculated that HOX proteins might function as regulators to mediate cellular proliferation in the skin and play vital roles in the regeneration of skin wounds [7]. The *HOXC6* is a member of the HOXC group, which is located on chromosome 12 [13]. To date, studies on *HOXC6* have mainly focused on its roles in tumor proliferation, apoptosis, migration and invasion [13–16]. Based on the information provided above, we hypothesized that *HOXC6* may play a key role in keloid progression. This study is the first to explore the role of *HOXC6* in keloid pathology and development.

Accumulating evidence has indicated that keloids have a relatively hypoxic internal environment due to the microvessel occlusion [17,18] and the increased oxygen level required for rapid growth [19]. Under hypoxic conditions *in vitro*, keloid-derived keratinocytes show a partial fibroblast-like appearance and acquire an enhanced aggressive capacity [20]. Kang *et al*. also showed that a hypoxic microenvironment leads to increased accumulation of extracellular collagen in keloids [21]. These findings suggested that hypoxia is an integral factor in keloidogenesis and keloid development. As a core marker of hypoxia, hypoxia-inducible factor-1α (HIF-1α) was also observed to be expressed at high levels in keloids compared to normal skin [22]. HIF-1α promotes proliferation and collagen synthesis and inhibits apoptosis in KFs [19,22]. However, the specific mechanism by which HIF-1α promotes keloid development remains unclear. To the best of our knowledge, the relationship between *HIF-1α* and *HOXC6* has not been described previously. Accordingly, we also explored the effect of *HIF-1α* on KFs and its interaction with *HOXC6*.

In this study, we first screened the differentially expressed genes (DEGs) between KFs and normal skin fibroblasts (NFs) to explore potential biological targets in the development of keloid. These analyses identified *HOXC6* as the hub gene in two expression profile datasets from the Gene Expression Omnibus (GEO) database (GSE145725 and GSE44270). Given the lack of previous reports of the role of *HOXC6* in keloid, we further validated the significance of high *HOXC6* expression in keloid tissue samples and KFs and explored the potential pathogenic mechanism of *HIF-1α*-induced *HOXC6* expression in KFs.

## Methods

### Microarray data processing and identification of DEGs

We obtained two expression profile datasets of keloids, GSE145725 [21] and GSE44270 [23], from the GEO database (https://www.ncbi.nlm.nih.gov/geo/). Nineteen samples from GSE145725 (10 NFs and 9 KFs) and twelve samples from GSE44270 (3 NFs and 9 KFs) were selected for analysis in this study (Table 1). The original expression matrix was processed and analyzed using R software (version 4.0.2). DEGs were screened using the Limma package [24]. Genes with *p* < 0.05 and |log_2_foldchange (FC)| > 1 were defined as DEGs in GSE145725, whereas genes with *p* < 0.05 and |log_2_FC| > 0.5 were defined as DEGs in GSE44270. A volcano plot was constructed to visualize the data using Hiplot (https://hiplot.com.cn/). TB tools was used to construct a Venn diagram to identify common DEGs from the two datasets [25].

**Table 1:** Datasets information included in our study

**GEO accession**	**KF**	**NF**	**Number of samples**		**Platform**	**Time**
			**KF**	**NF**		
GSE145725	GSM4331585 GSM4331586 GSM4331587 GSM4331588 GSM4331589 GSM4331590 GSM4331591 GSM4331592 GSM4331593	GSM4331594 GSM4331595 GSM4331596 GSM4331597 GSM4331598 GSM4331599 GSM4331600 GSM4331601 GSM4331602 GSM4331603	9	10	GPL16043	2020
GSE44270	GSM1081582 GSM1081583 GSM1081584 GSM1081585 GSM1081586 GSM1081587 GSM1081588 GSM1081589 GSM1081590	GSM1081608 GSM1081609 GSM1081610	9	3	GPL6244	2013

*GEO* Gene Expression Omnibus, *KF* keloid fibroblasts, *NF* normal skin fibroblasts, *GSE* gene set ensemble, *GSM* gene set matrix, *GPL* GEO platform

### Enrichment analysis

Gene ontology (GO) enrichment analysis of the common DEGs in KFs from the GEO datasets was performed using the clusterProfile package [26] of R software and further visualized using Hiplot. GO terms were selected at a cut-off of *p* < 0.05.

Genetic information of KFs and NFs from GEO was uploaded to perform a gene set enrichment analysis (GSEA) [27], which ranks genes based on the degree of differential expression between two groups, followed by testing whether the gene set is significantly enriched at the top or bottom of the ranking list. Rather than focusing on individual genes, this analysis evaluates the expression of the whole genome, which enables the inclusion and analysis of genes with more subtle changes.

### Protein–protein interaction analysis

DEGs were uploaded to STRING (http://string-db.org) to construct a protein–protein interaction (PPI) network and provide insights into the mechanisms of keloid pathogenesis [28]. The minimum required interaction score was set to a medium confidence level (0.400). CytoHubba, a Cytoscape plugin, was used to rank the top nine genes as hub genes based on their degree value in the network [29, 30].

### Clinical specimens

Keloid tissue specimens and normal skin specimens were excised at the Department of Dermatology, Nanfang Hospital, Southern Medical University. Keloid was diagnosed based on clinical history, clinical manifestation and histopathologic examination. Patients were excluded if their keloids had been exposed to glucocorticoids, 5-fluorouracil, botulinum toxin type A, cryosurgery, radiation or laser therapies. Normal skin tissues were collected from healthy patients undergoing cosmetic surgery at the Dermatology Clinic. Seven keloid tissues and five normal skin tissues were collected and stored at −80°C until analysis using reverse transcription-quantitative polymerase chain reaction (RT-qPCR), and five keloid tissues and five normal skin tissues were preserved for subsequent western blot (WB) analysis. Five keloid tissues and five normal skin tissues were stored in formalin for immunohistochemistry. Seven keloid tissue specimens and seven normal skin specimens were used to establish primary cell lines. The Ethics Committee of Nanfang Hospital, Southern Medical University approved this study. All patients signed written informed consent forms.

### Primary fibroblast culture

The epidermis and subcutaneous adipose tissues were removed from the specimens. The dermal tissues were cut into 3 × 3 mm sections and allowed to attach to the culture flask. Dulbecco’s modified Eagle’s medium (DMEM) supplemented with 10% fetal bovine serum (FBS) was added to the flask, and the fibroblasts were cultured at 37°C in an atmosphere of 5% CO_2_. Subculture was performed after the cultured cells reached 70–80% confluence. NFs and KFs were used at passages 3–5 for experiments.

We added the extracellular regulated protein kinase (ERK) inhibitor SCH772984 (Selleckchem, Houston, USA) to KFs at the indicated concentrations to observe the effect of ERK on KFs and NFs.

### Cell transfection

*HOXC6*, *HIF-1α* and negative control small interfering RNAs (siRNAs) were designed and synthesized by Ribobio (Guangzhou, China). Since the effectiveness of siRNAs varies, we designed three different siRNA sequences each for *HOXC6* and *HIF-1α*, and chose the sequence with the best interference efficiency (Table 2). NFs and KFs were seeded in 6-well plates at a density of 1 × 10^5^ cells/well and cultured with DMEM/10% FBS until they reached 50–60% confluence. The KFs were then separately transfected with *HOXC6* siRNAs (si-*HOXC6*-KF), *HIF-1α* siRNAs (si-*HIF-1α*-KF) and a negative control siRNA (si-NC-KF) using Lipofectamine 2000 transfection reagent (Life Technologies, Invitrogen, Carlsbad, CA, USA) and Opti-MEM (11058–021, Gibco, Grand Island, NY, USA) according to the manufacturers’ protocols. The NFs were transfected with si-NC (si-NC-NF) according to the manufacturers’ protocols. Six hours after transfection, fresh medium was added and the cells were incubated for 48 h. A fluorescence microscopy inspection and RT-qPCR were used to analyze the interference efficiency of the siRNAs.

**Table 2:** Sequences used in this study

**Gene**	**Primer (5′–3′)**
si-HOXC6 si-HIF-1α	GCGAATGAATTCGCACAGT CTACTCAGGACACAGATTTAGACTTGGAG
HOXC6	F: CCAGAAAGCCAGTATCCAGAT
	R: TCTGGTACCGCGAGTAGATC
Collagen I	F: CCTGGATGCCATCAAAGTCT R: ACTGCAACTGGAATCCATCG
α-SMA	F: CCTTGAGAAGAGTTACGAGTTG R: TGCTGTTGTAGGTGGTTTCA
GAPDH	F: CTCTGACTTCAACAGCGACACC
	R: CTGTTGCTGTAGCCAAATTCGTT
18 S	F: CCTGGATACCGCAGCTAGGA R: GCGGCGCAATACGAATGCCCC
The promoter of HOXC6	F: cggggtaccCAAATGCTCCCGCCGTCCCATTACCGGAATG R: ctagctagcTATTAATCAGATTAATTTAAAAAAAATACTGC

*si-HOXC6* HOXC6 siRNA, *si-HIF-1α* HIF-1α siRNA, *F* forward sequence, *R* reverse sequence, *HOXC6* homeobox C6, *HIF-1α* hypoxia-inducible factor-1α, *α-SMA* α-smooth muscle actin, *GAPDH* glyceraldehyde-3-phosphate dehydrogenase

### RT-qPCR analysis

Total RNA was extracted from each specimen using TRIzol (Takara Bio Inc., Shiga, Japan). The concentration and purity of the total RNA were assessed using an ND 1000 microspectrophotometer (Nanodrop; Thermo Fisher Scientific Inc., Wilmington, DE, USA). The RT reaction was conducted using a PrimeScript™ RT reagent kit (RR037A, Takara Bio Inc., Shiga, Japan) at 37°C for 15 min and 85°C for 5 s, followed by storage at 4°C. Next, RT-qPCR was performed with TB Green® Premix Ex Taq™ II (RR820A; Takara Bio Inc.) and the QuantStudio 6 Flex Real-Time PCR System (Applied Biosystems Inc., Carlsbad, CA, USA). *Glyceraldehyde-3-phosphate dehydrogenase* (*GAPDH*) or *18S* was used as the internal control gene. The 2^−ΔΔCt^ method was used to determine the relative expression of the target mRNAs. The primer sequences used in this study are shown in Table 2.

### Immunohistochemistry

Immunohistochemical staining was performed to semiquantitatively evaluate the HOXC6 protein levels in keloid tissues. The sections were deparaffinized in xylene and dehydrated with an ethanol gradient, followed by heating in a microwave oven at a controlled final temperature of 121°C for 15 min. A primary antibody against HOXC6 (anti-HOXC6; ab41587, Abcam Inc., Cambridge, MA, USA) was used at a 1:200 dilution. After incubation with 3,3′-diaminobenzidine (DAB) liquid for 1 min, the slides were counterstained with Mayer’s hematoxylin. The presence of light yellow or tan staining in the cytoplasm was regarded as a positive result. The histoscore (H-score) was applied to KFs and NFs in the dermis, where both the percentage and the intensity of the positive staining were considered. H-score = 1 × the percentage of mildly stained fibroblasts +2 × the percentage of moderately stained fibroblasts +3 × the percentage of strongly stained fibroblasts [31].

### WB analysis

Proteins were extracted from fibroblasts or tissues using lysis buffer (Beyotime) supplemented with 1 mM phenylmethanesulfonyl fluoride. A bicinchoninic acid protein assay kit (KGPBCA, Jiangsu KeyGEN BioTECH Corp., Ltd, Jiangsu, China) was used to detect the protein concentrations. Equal amounts of protein (20 μg) were separated by 12% sodium dodecyl sulfate–polyacrylamide gel electrophoresis and transferred to polyvinylidene difluoride membranes (Millipore, Boston, MA, USA) for immunoblotting. Primary antibodies against HOXC6, collagen I (66761–1-Ig, Proteintech, Chicago, USA), ERK (A5029, Bimake, USA) and HIF-1α (20960–1-AP, Proteintech, Chicago, USA) were used at a 1:2000 dilution. Primary antibodies against α-smooth muscle actin (α-SMA) (55135–1-AP, Proteintech, Chicago, USA) and p-ERK (A5036, Bimake, USA) were used at a 1:1000 dilution. The GAPDH primary antibody (AF7021, Affinity Biosciences, Jiangsu, China) was used as the internal reference. After incubation with the appropriate horseradish peroxidase-conjugated secondary antibodies, the immune complexes were measured using enhanced chemiluminescence (WBKLS0500, Millipore, USA).

### Cell proliferation assay

Cell proliferation was assessed using a Cell Counting Kit-8 (CCK-8, Beyotime, Shanghai, China) according to the manufacturer’s instructions. si-NC-NF, si-NC-KF, si-HOXC6-KF, NFs, KFs and KFs treated with SCH774982 (KF + SCH774982) were seeded in 96-well plates at a density of 1 × 10^4^ fibroblasts per well. Three parallel wells were used for each group. CCK-8 reagent (10 μL) was added to each well after fibroblasts were incubated for 0, 12, 24 and 72 h at 37°C under a humidified atmosphere of 5% CO_2_. The fibroblasts were then incubated for another 30 min. The absorbance of each sample was recorded at 450 nm using a microplate reader (Thermo Fisher Scientific, Inc., Pittsburgh, PA, USA).

### Cell apoptosis assay

A fluorescein isothiocyanate (FITC) Annexin V Apoptosis Detection Kit I (556 547, BD Bioscience, Franklin Lakes, NJ, USA) was used for the cell apoptosis assay. Fibroblasts from different groups were seeded in six-well plates at a density of 1 × 10^6^ fibroblasts per well. The fibroblasts were double stained according to the manufacturer’s instructions. A 100 μL cell suspension was added to a test tube and stained with 5 μL of FITC-Annexin V and 5 μL of propidium iodide (PI) for 15 min at 25°C in the dark. Data acquisition and analysis were performed using a BD FACSCanto II flow cytometer (Becton Dickinson, USA).

### Cell migration assay

Cell migration was assessed using Transwell chambers with a transparent PET membrane with an 8 μm pore size (353 097, BD Falcon, BD Biosciences, Bedford, USA). Fibroblasts were seeded in six-well plates (1 × 10^5^ fibroblasts per well) and incubated for 24 h. Then, fibroblasts in serum-free DMEM were seeded in the upper chambers, and the lower chambers were filled with the same medium supplemented with 10% FBS. After 48 h of incubation, the nonmigrated fibroblasts remaining on the upper surface of the membrane were removed with cotton swabs. Fibroblasts that migrated to the lower chambers were fixed with 4% paraformaldehyde for 10 min and stained with ready-to-use crystal violet for 10 min. The number of migrated fibroblasts in three randomly selected fields was counted under an inverted microscope. The experiments were performed in triplicate.

### Dual luciferase reporter assay

The JASPAR database (http://jaspar.genereg.net/) was used to predict the binding sites for *HIF-1α* in the *HOXC6* promoter region, and a dual-luciferase reporter assay was conducted to confirm the targeting relationship between *HIF-1α* and *HOXC6*. 293 T cells (GuangZhou Jennio Biotech Co., Ltd, Guangzhou, China) were seeded in 12-well plates with complete medium for 24 h before transfection. The *HOXC6* promoter sequence was amplified using the primers shown in Table 2. The *HOXC6* promoter luciferase reporter vector (Wt-pGL3-HOXC6) was generated after the target fragment was recovered by agarose gel electrophoresis and was ligated between the KpnI and NheI restriction sites of the pGL-3 basic vector. Two specific primers were designed and synthesized by GENEWIZ, Inc. (Suzhou, China), and a mutant vector of the *HOXC6* promoter (Mut-pGL3-HOXC6) was constructed according to the instructions of the site-specific mutation kit, in which the three predicted binding sites were knocked out at the same time. The *HIF-1α* overexpression vector (pcDNA-*HIF-1α*) and the negative control vector (pcDNA-Empty) were also constructed. The *Renilla* luciferase expression vector pGL-3Basic (Promega, Madison, WI, USA) served as the internal reference. pcDNA-*HIF-1α* and the luciferase reporter vector were cotransfected into 293 T cells with Lipofectamine 2000 (11 668 030, Thermo Fisher, USA), and luciferase activity was measured using a dual-luciferase reporter assay system (Promega, USA) 48 h later. Relative luciferase activity was calculated.

**Figure 1.**
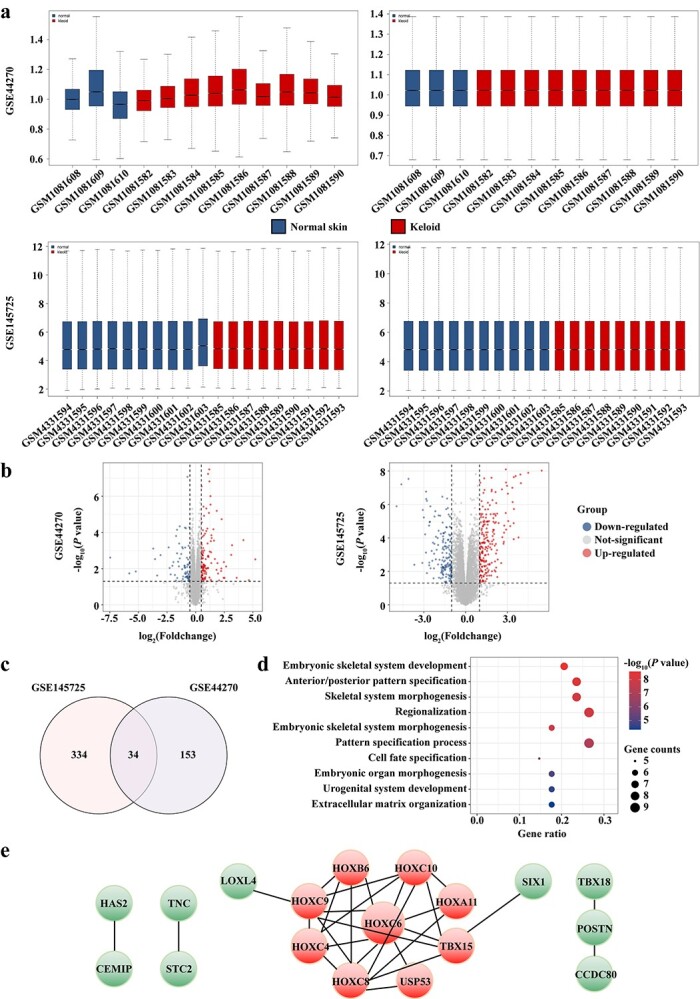
*HOXC6* was identified as the top-ranking hub gene of KFs in microarray datasets. (**a**) Boxplot of GSE44270 and GSE145725 before normalization and after normalization. (**b**) Volcano plot of the distributions of all DEGs of GSE44270 (*p* < 0.05, |log_2_FC| >0.5) and GSE145725 (*p* < 0.05, |log_2_FC| > 1). Blue dots represent significantly down-regulated genes and red dots represent significantly up-regulated genes in KFs samples. (**c**) Venn plot of common DEGs identified between two data sets. (**d**) Top ten enriched GO biological process terms. (**e**) PPI network of the DEGs in two data sets. The nodes indicate the DEGs and the edges indicate the interactions between two proteins. Medium confidence score was used for the construction of PPI networks. Red dots represent the top nine hub genes with degree by CytoHubba plugin. *HOXC6* homeobox C6, *KFs* keloid fibroblasts, *GSE* gene set ensemble, *GSM* gene set matrix, *FC* fold change, *DEGs* differentially expressed genes, *GO* gene ontology, *PPI* protein–protein interaction

**Table 3:** GO enrichment analyses of DEGs from GSE145725 and GSE44270

**Category**	**GO ID**	**GO Terms**	***P* value**	**Count**
Biological process	GO:0048706	Embryonic skeletal system development	2.49E-09	7
Biological process	GO:0009952	Anterior/posterior pattern specification	4.41E-09	8
Biological process	GO:0048705	Skeletal system morphogenesis	8.74E-09	8
Biological process	GO:0003002	Regionalization	9.18E-09	9
Biological process	GO:0048704	Embryonic skeletal system morphogenesis	1.56E-08	6
Biological process	GO:0007389	Pattern specification process	7.22E-08	9
Biological process	GO:0001708	Cell fate specification	9.33E-07	5
Biological process	GO:0048562	Embryonic organ morphogenesis	1.20E-05	6
Biological process	GO:0001655	Urogenital system development	2.58E-05	6
Biological process	GO:0030198	Extracellular matrix organization	4.75E-05	6
Molecular function	GO:0001228	DNA-binding transcription activator activity, RNA polymerase II-specific	1.06E-03	5
Molecular function	GO:0001216	DNA-binding transcription activator activity	1.07E-03	5
Molecular function	GO:0033613	Activating transcription factor binding	4.80E-04	3
Cellular component	GO:0005614	Interstitial matrix	1.88E-04	2

*GO* gene ontology, *DEGs* differentially expressed genes, *ID* identification

### Transcriptome sequencing and bioinformatics analysis

Transcriptome sequencing was performed to compare the downstream signaling pathways of *HOXC6* between the si-*HOXC6*-KF group (*n* = 3) and the si-NC-KF group (*n* = 3), which was implemented by Novogene Ltd (Beijing, China). Total RNA was extracted using TRIzol (Takara Bio Inc., Shiga, Japan) and the quality and quantity of the isolated RNA were assessed using an ND 1000 microspectrophotometer (Nanodrop; Thermo Fisher Scientific Inc., Wilmington, DE, USA). The RNA library was constructed using an NEBNext® Ultra™ RNA Library Prep Kit (Illumina, Inc., San Diego, USA) according to the manufacturer’s instructions. DESeq2 R software (version 1.32.0) (http://bioconductor.org/packages/release/bioc/html/DESeq2.html) [32] was used to identify the DEGs according to the screening criteria of *p* ≤ 0.05 and |log_2_FC| ≥ 0 and to predict the underlying regulatory mechanisms of *HOXC6* in KFs. The boxplot, the heatmap and the volcano plot were visualized using the R software. A Reactome analysis of the DEGs was performed using clusterProfiler package of R software (version 4.0.2) (http://bioconductor.org/packages/release/bioc/html/clusterProfiler.html) [26,33], and the results are presented as a bubble chart and a histogram constructed with the R ggplot2 package.

### Statistical analysis

The data obtained in this study were compared using Student’s *t-*test or one-way ANOVA. All statistical analyses were performed using SPSS software (version 25.0; IBM Corp., Armonk, NY, USA). A *p* value <0.05 was considered statistically significant.

## Results

### *HOXC6* was identified as the top-ranking hub gene of KFs in microarray datasets

Based on the sample information and data matrix, 368 and 187 genes were found to be differentially expressed between the KF and NF samples from GSE145725 (*p* < 0.05, |log_2_FC| > 1) and GSE44270 (*p* < 0.05, |log_2_FC| >0.5), respectively. The boxplots of gene expression before and after normalization are presented in Figure 1a and the volcano plot is shown in Figure 1b. A total of 34 common DEGs from these two datasets intersected (Figure 1c).

In terms of biological processes, the GO enrichment analysis of the 34 common DEGs showed that the gene sets were significantly enriched in embryonic skeletal system development, anterior/posterior pattern specification, skeletal system morphogenesis, regionalization, embryonic skeletal system morphogenesis, pattern specification process, cell fate specification, embryonic organ morphogenesis, urogenital system development and ECM organization (Figure 1d). Molecular function analysis revealed that the DEGs were associated with activating transcription factor binding, RNA polymerase II specific DNA-binding transcription activator activity and DNA-binding transcription activator activity. The cellular components of DEGs were mainly enriched in the interstitial matrix (Table 3). In total, 17 nodes plus 29 edges were obtained to identify the core genes among the DEGs (Figure 1e). Nine hub genes, *HOXC6*, *HOXC8*, *HOXC9*, *HOXC10*, *HOXC4*, *TBX15*, *HOXB6*, *HOXA11* and *USP53* (Figure 1e, red dots), were identified by PPI analysis. Among these genes, HOXC6 had the highest degree in the PPI network and was therefore selected as a candidate gene associated with keloid pathogenesis for further confirmation.

**Figure 2.**
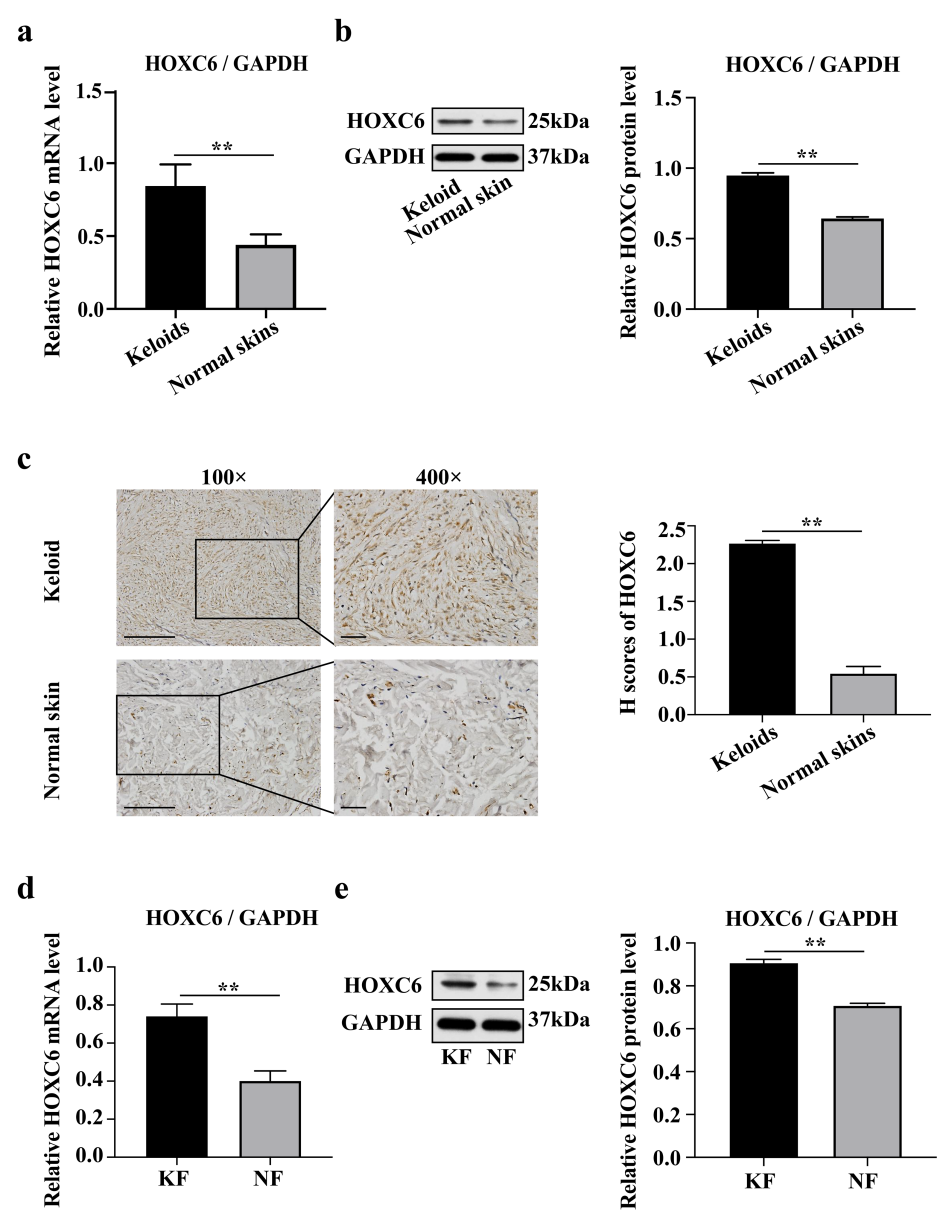
HOXC6 is upregulated in keloid tissues and fibroblasts. (**a**) mRNA expression of *HOXC6* in keloid tissues and normal skin tissues was determined by RT-qPCR (*n* = 5). *GAPDH* served as an internal control. (**b**) Protein expression of HOXC6 in keloid tissues and normal skin tissues was assessed by western blot (*n* = 5). GAPDH served as a loading control. (**c**) Protein expression of HOXC6 in keloid tissues and normal skin tissues was shown by immunohistochemical staining (*n* = 5). Scale bar = 100 μm (×100 magnification) and 50 μm (×400 magnification), respectively. (**d**) mRNA expression of *HOXC6* in KF and NF was determined by RT-qPCR (*n* = 3). *GAPDH* served as an internal control. (*e*) Protein expression of HOXC6 in KF and NF was detected by western blot (*n* = 3). GAPDH served as a loading control. Data are representative images or expressed as mean ± SD. ^*^^*^p < 0.01. *HOXC6* homeobox C6, *KF* keloid fibroblasts, *NF* normal skin fibroblasts, *mRNA* messenger RNA, *GAPDH* glyceraldehyde-3-phosphate dehydrogenase, *H-scores* histoscores

**Figure 3.**
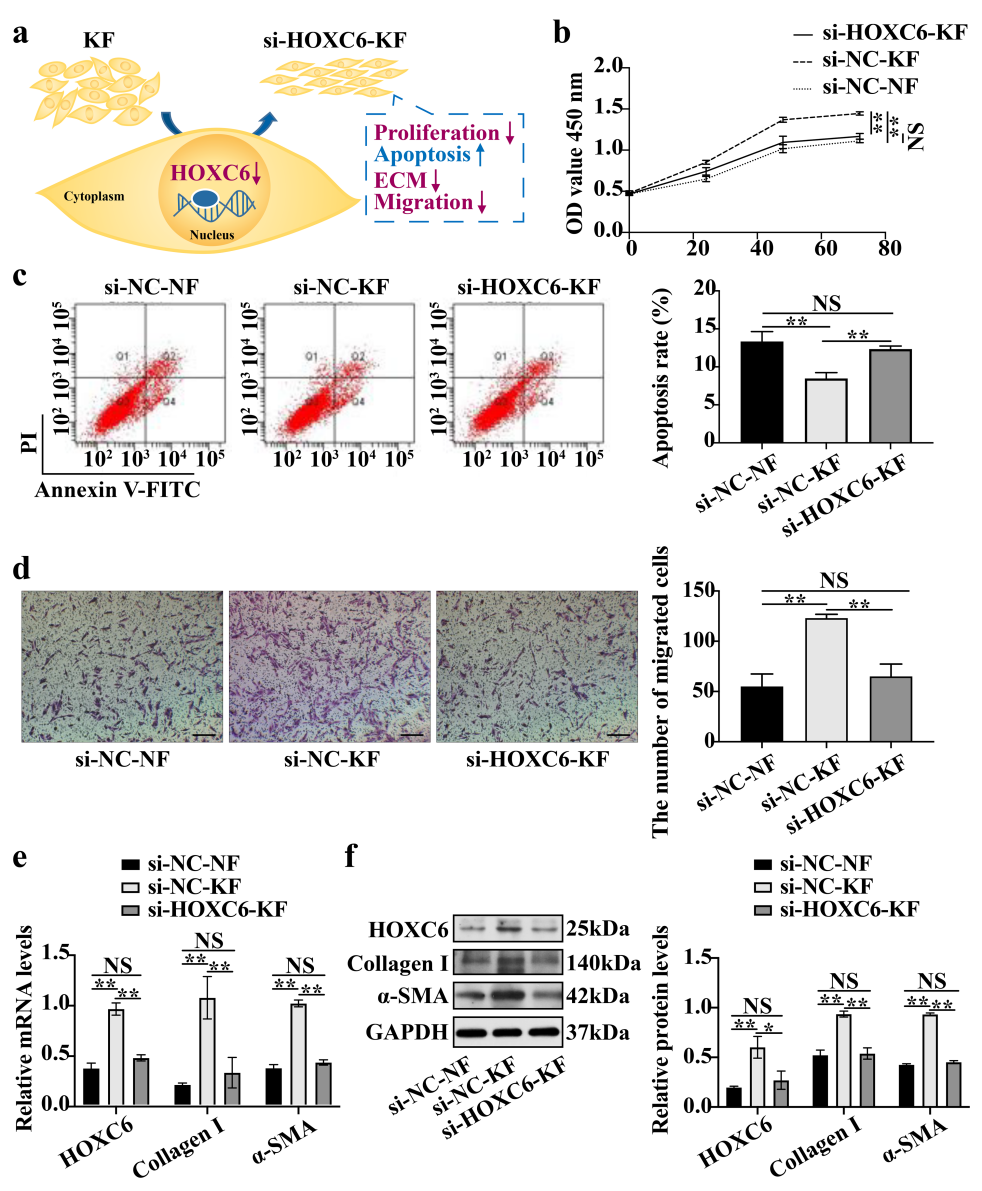
Downregulation of *HOXC6* inhibits proliferation, migration, ECM accumulation and promotes apoptosis in keloid fibroblasts. (**a**) Schematic representation of the main process of down-regulated *HOXC6* suppressing proliferation, apoptosis inhibition, migration and ECM accumulation in keloid fibroblasts. (**b**) Proliferation of si-*HOXC6*-KF was assessed by CCK-8 assay. (**c**) Apoptosis of si-*HOXC6*-KF was monitored by flow cytometry. (**d**) Migration of si-*HOXC6*-KF was detected by transwell assay. Scale bar = 100 μm (×20 magnification). (**e**) mRNA expression of *collagen I* and *α-SMA* in si-*HOXC6*-KF was detected by RT-qPCR. *18S* served as an internal control. (**f**) Protein expression of collagen I and α-SMA in si-*HOXC6*-KF was detected by western blot. GAPDH served as a loading control. Data are representative images or expressed as the means ± SD (*n* = 3). ^*^p < 0.05; ^*^^*^p < 0.01. *NS* non-significant, *HOXC6* homeobox C6, *si-HOXC6-KF* keloid fibroblasts transfected with HOXC6 siRNAs, *si-NC-KF* keloid fibroblasts transfected with the negative control siRNAs, *si-NC-NF* normal skin fibroblasts transfected with the negative control siRNAs, *OD* optical density, *α-SMA* α*-*smooth muscle actin, *ECM* extracellular matrix, *GAPDH* glyceraldehyde-3-phosphate dehydrogenase, *mRNA* messenger RNA

**Figure 4.**
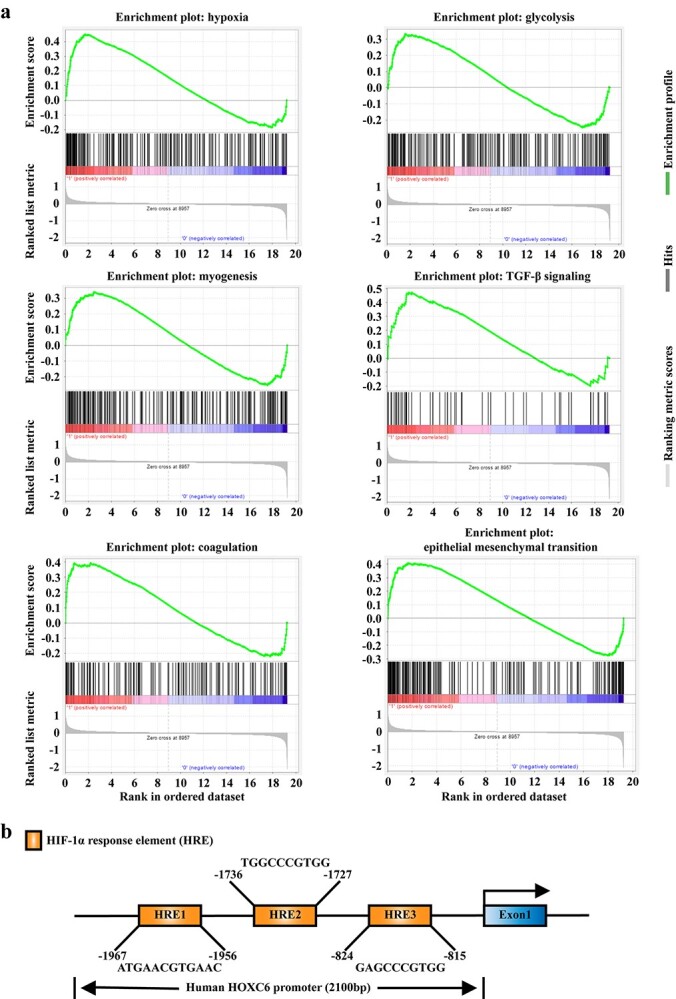
*HIF-1α* is predicted to be involved in the pathways upstream of *HOXC6* in keloid fibroblasts. (**a**) The critical pathways associated with HOXC6 in keloid fibroblasts were explored by gene set enrichment analysis. (**b**) HIF-1α binding sites on the promoter region of HOXC6 were predicted by JASPAR database. *HOXC6* homeobox C6, *HIF-1α* hypoxia-inducible factor-1α, *HRE* HIF-1α response element

### HOXC6 is upregulated in keloid tissues and KFs

RT-qPCR, WB and immunohistochemistry analyses were performed to confirm the expression of HOXC6 in keloids. Consistent with predictions, the *HOXC6* mRNA level was significantly higher in keloid tissues than in normal skin tissues (Figure 2a). Moreover, immunohistochemistry revealed stronger HOXC6 expression in KFs than in NFs (Figure 2c), which was further confirmed and quantified using WB analysis (Figure 2b). In parallel, we examined HOXC6 levels in KFs. Consistently, HOXC6 mRNA and protein expression levels were significantly upregulated in KFs compared with NFs (Figure 2d, e). Based on the aforementioned results, we speculated that HOXC6 may represent a potentially new biomarker for keloid development.

### Downregulation of *HOXC6* inhibits proliferation, migration and ECM accumulation and promotes apoptosis in KFs

We explored the effects of *HOXC6* downregulation on cell proliferation, migration, apoptosis and ECM synthesis to determine the function of high HOXC6 expression in keloids and KFs (Figure 3a). The CCK-8 assay showed a noticeable decrease in the proliferation of the si-*HOXC6*-KF group compared with that of the si-NC-KF group (Figure 3b). FITC-Annexin V/PI staining further showed that *HOXC6* downregulation markedly increased the proportion of apoptotic cells among KFs (Figure 3c). The transwell assay showed that the number of migrated si-*HOXC6*-KFs was significantly decreased compared with that of si-NC-KFs (Figure 3d). ECM accumulation is a well-characterized process that contributes to the growth and invasion of keloids. Two known ECM-related biomarkers, collagen I and α-SMA, were detected using RT-qPCR and WB analyses to determine the effects of HOXC6 on ECM production by KFs. Levels of the collagen I and α-SMA mRNAs and proteins were downregulated in the si-*HOXC6*-KF group compared with the si-NC-KF group (Figure 3e, f). Notably, no significant difference was observed between the si-*HOXC6*-KF group and the si-NC-NF group (Figure 3b-f).

**Figure 5.**
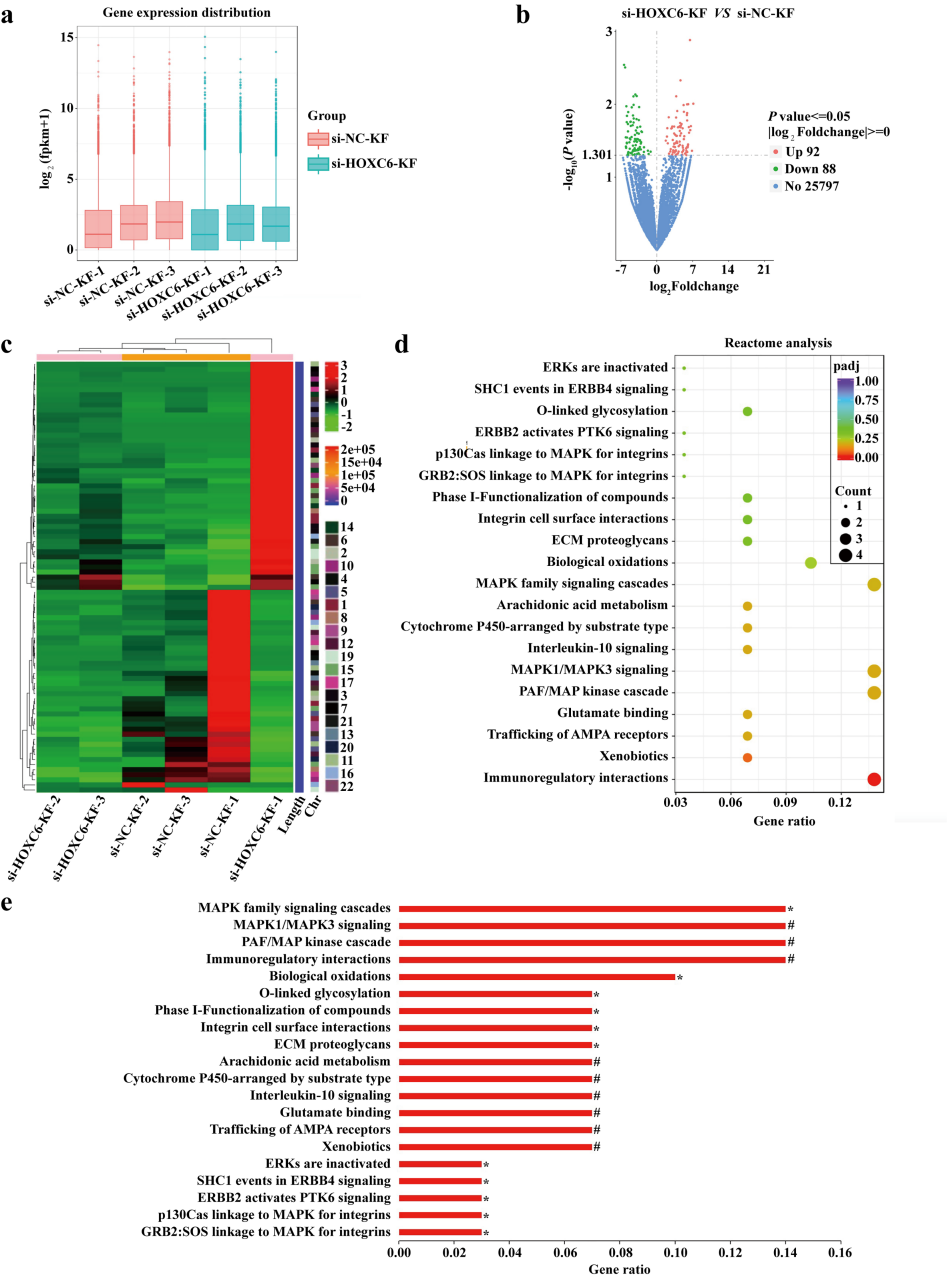
The ERK pathway is predicted to be involved in the pathways downstream of HOXC6 in keloid fibroblasts. (**a**) Boxplot of the si-*HOXC6*-KF group and the si-NC-KF group. (**b**) Volcano plot of the distributions of all DEGs between the si-*HOXC6*-KF group and the si-NC-KF group, with red dots representing 92 significantly up-regulated genes and green dots representing 88 significantly down-regulated genes in the si-*HOXC6*-KF group. No significantly changed genes are marked as blue dots (25797). (**c**) Heatmap of si-*HOXC6*-KF group and si-NC-KF group. (**d**) Scatter diagram of the top twenty signaling pathways based on Reactome analysis. (**e**) Sort the top twenty pathways in Reactome Pathway analysis by GeneRatio. ^*^p < 0.05; #*p* < 0.01. *HOXC6* homeobox C6, s*i-HOXC6-KF* keloid fibroblasts transfected with HOXC6 siRNAs, *si-NC-KF* keloid fibroblasts transfected with the negative control siRNAs, *fpkm* fragments per kilobase of exon model per Million mapped fragments, *padj p*-Value adjusted, *MAPK* mitogen-activated protein kinase, *ERK* extracellular regulated protein kinase, *AMPA* α-amino-3-hydroxy-5-methyl-4-isoxazole-propionicacid, *MAP* mitogen-activated protein, *PAF* platelet activating factor, *ECM* extracellular matrix, *GRB2* growth factor receptor-bound protein-2, *ERBB4* receptor protein-tyrosine kinase erbB-4, *PTK6* protein tyrosine kinase 6, *SHC1* SHC transforming protein 1

**Figure 6.**
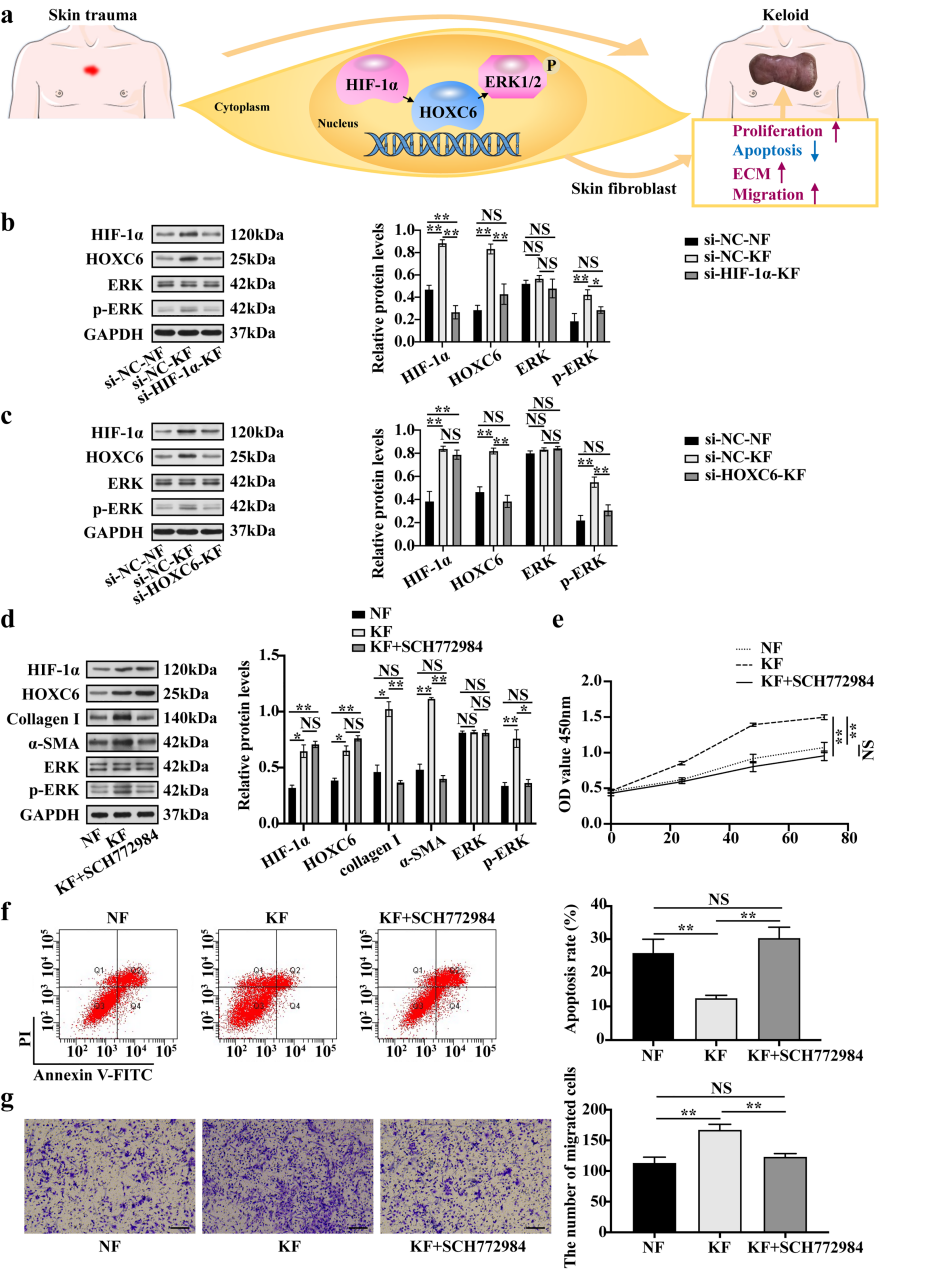
HIF-1α-induced HOXC6 promotes the development of keloid fibroblasts through the ERK signaling pathway. (**a**) Schematic representation of the HIF-1α/HOXC6/ERK axis involved in keloid fibroblasts. HIF-1α positively regulates HOXC6, which further promotes the proliferation, migration, apoptosis inhibition and ECM accumulation of keloid fibroblasts through the ERK signaling pathway. HOXC6 could be a promising novel therapeutic target in keloid. (**b**) Protein expression of HIF-1α, HOXC6 and p-ERK in the si-HIF-1α-KF group was assessed by western blot. GAPDH served as a loading control. (**c**) Protein expression of HIF-1α, HOXC6 and p-ERK in the si-HOXC6-KF group was assessed by western blot. GAPDH served as a loading control. (**d**) Protein expression of HIF-1α, HOXC6, collagen I, α-SMA and p-ERK in the KF + SCH772984 group was assessed by western blot. GAPDH served as a loading control. (**e**) Proliferation of the KF + SCH772984 group was assessed by CCK-8 assay. (**f**) Apoptosis of the KF + SCH772984 group was monitored by flow cytometry. (**g**) Migration of cells in the KF + SCH772984 group was detected by transwell assay. Scale bar = 100 μm (×20 magnification). Data are representative images or expressed as the means ± SD (*n* = 3). ^*^p < 0.05; ^*^^*^p < 0.01. *NS* non-significant, *HOXC6* homeobox C6, *HIF-1α* hypoxia-inducible factor-1α, *ERK* extracellular regulated protein kinase, *p-ERK* phosphorylated extracellular regulated protein kinase, *si-HIF-1α-KF* keloid fibroblasts transfected with HIF-1α siRNAs, *si-HOXC6-KF* keloid fibroblasts transfected with HOXC6 siRNAs, *KF + SCH772984* keloid fibroblasts treated with SCH772984, *si-NC-KF* keloid fibroblasts transfected with the negative control siRNAs, *si-NC-NF* normal skin fibroblasts transfected with the negative control siRNAs, *KF* keloid fibroblasts, *NF* normal skin fibroblasts, *α-SMA* α*-*smooth muscle actin, *GAPDH* glyceraldehyde-3-phosphate dehydrogenase

### *HIF-1α* is predicted to be involved in the pathways upstream of *HOXC6* in KFs

The GSEA database was used to predict the possible underlying mechanisms of *HOXC6* in KFs. *HOXC6*-related pathways in KFs were enriched in hypoxia, TGF-β, glycolysis, epithelial–mesenchymal transition, coagulation and myogenesis signaling (Figure 4a). The hypoxic stress response was predicted to be the main *HOXC6*-related pathway in KFs.

Since HIF-1α is a core factor that mediates hypoxic stress and plays a key role in keloid development [22], the JASPAR database was used to forecast the possible *HIF-1α* binding sites within the *HOXC6* promoter region. The results indicated three binding sites for *HIF-1α* within the 2100 bp promoter region of *HOXC6* (Figure 4b).

The dual-luciferase reporter assay revealed that *HIF-1α* promotes the expression of *HOXC6*, but the *HIF-1α* overexpression vector increased the luciferase activity of the wild-type promoter sequence and the mutant promoter sequence of *HOXC6* (supplementary Fig. S1, see online supplementary material).

### The ERK pathway is predicted to be involved in the pathways downstream of HOXC6 in KFs

Three pairs of si-*HOXC6*-KF and si-NC-KF groups were used to conduct a transcriptome sequencing experiment, followed by a series of bioinformatics analyses to explore the possible pathways downstream of *HOXC6* in KFs. The boxplot and heatmap of the data matrix are presented in Figure 5a and c, respectively. As shown in the volcano plot (Figure 5b), 180 genes were significantly differentially expressed in the two groups, including 92 upregulated genes and 88 downregulated genes. The Reactome enrichment analysis (Figure 5d, e) suggested that the ERK/mitogen-activated protein kinase (MAPK)-related signaling pathways were significantly enriched in the DEGs, including MAPK1/MAPK3 signaling, MAPK family signaling cascades, ERK inactivation, p130Cas linkage to MAPK for integrins and growth factor receptor-bound protein-2:SOS linkage to MAPK for integrins.

### HIF-1α-induced HOXC6 promotes the development of KFs through the ERK signaling pathway

We conducted a series of experiments to explore the role of the HIF-1α/HOXC6/ERK axis in KFs (Figure 6a). WB analyses showed prominent decreases in the levels of the HIF-1α, HOXC6 and p-ERK proteins in the si-*HIF-1α*-KF group compared with the si-NC-KF group, but differences in the levels of the HOXC6 and p-ERK proteins between the si-*HIF-1α*-KF group and the si-NC-NF group were not significant (Figure 6b). WB analyses also showed remarkably lower levels of the HOXC6 and p-ERK proteins in the si-*HOXC6*-KF group. A significant difference was not observed between the si-*HOXC6*-KF group and the si-NC-NF group (Figure 6c).

Compared with the KF group, levels of the collagen I, α-SMA and p-ERK proteins were obviously reduced in the KF + SCH772984 group, while a significant difference was not observed between the KF + SCH772984 group and the NF group (Figure 6d). The CCK-8 assay showed that the proliferation of the KF + SCH772984 group evidently decreased compared with that of the KF group (Figure 6e). The apoptosis assay showed that the proportion of apoptotic cells among the KF + SCH772984 group significantly increased (Figure 6f). Compared with the KF group, the transwell assay showed that the number of migrated cells in the KF + SCH772984 group was significantly reduced (Figure 6g). No significant difference was observed in fibroblast proliferation, apoptosis rate, migration ability or ECM deposition between the KF + SCH772984 group and the NF group (Figure 6e–g).

## Discussion

In this study, we first identified a crucial role for *HOXC6* in the pathogenic mechanism of KFs in keloids and documented that *HIF-1α* positively regulates *HOXC6* expression in KFs, thereby contributing to the progression of keloids. Initially, we identified *HOXC6* as the top-ranking hub gene of KFs in GEO datasets. Consistently, elevated mRNA and protein expression levels of HOXC6 were observed in keloid tissue samples and KFs. *HOXC6* knockdown inhibited proliferation, migration and ECM accumulation but promoted KF apoptosis. In addition, a series of bioinformatics analyses and *in vitro* experiments revealed that *HIF-1α* is an upstream positive regulator of *HOXC6*, and the ERK/MAPK pathway was downstream of *HIF-1α*-induced *HOXC6* expression in KFs, implying a vital role for *HOXC6* in the HIF-1α/HOXC6/ERK axis in the progression of keloids.

Comprised of four paralogous clusters (A, B, C and D), *HOX* genes are well-conserved transcription factors that regulate embryonic development and cellular morphogenesis and differentiation [34]. In addition to their role in embryonic development, *HOX* genes play important roles in several cellular processes, such as proliferation, differentiation, morphogenesis and cell–cell and cell–ECM interactions [35–37]. Recent studies have also suggested that *HOX* genes might also be involved in keloids. Hahn *et al*. [38] found that *HOX* genes, including *HOXA7, HOXA9*, *HOXC8* and *HOXC11*, are implicated in the development of keloids. HOXA11-AS, which transcriptionally regulates *HOXA11*, was also suggested to facilitate keloid formation by inducing collagen I production [39].

*HOXC6* functions as an oncogene in various tumors. *HOXC6* was reported to be overexpressed in colorectal cancer [40] and was associated with shorter survival of patients with hepatocellular carcinoma [41]. These findings indicated an important role for *HOXC6* in the pathology of aggressive tumors. Although keloids are histologically benign, they clearly share certain characteristics of nonmetastatic aggressive malignancies. However, the role of *HOXC6* and its specific mechanism in keloids remain unclear. As the main pathological cells in keloids, KFs display invasive proliferation and abnormal deposition of ECM [42]. Based on our results, *HOXC6* was overexpressed in keloid tissue samples and KFs, which subsequently promoted KF proliferation and migration and inhibited KF apoptosis. Accumulating evidence has shown that abnormal accumulation of collagens and other ECM components, such as α-SMA, contribute to the formation of keloids [43]. We found that *HOXC6* downregulation alleviated ECM accumulation in KFs. Thus, *HOXC6* might facilitate proliferation, migration and ECM deposition but suppress KF apoptosis.

This prominent keloidogenesis function of *HOXC6* encouraged us to further explore its upstream and downstream molecular mechanisms. By performing a GSEA, we found that the hypoxic stress response was a *HOXC6*-related pathway in KFs. According to the ischaemic hypothesis, keloids exist in a relatively hypoxic environment that contributes to their proliferation, migration and excess collagen deposition [17]. As an important oxygen sensor, HIF-1α is a transcription factor that is upregulated under hypoxic conditions [21]. Numerous studies have suggested a significantly higher HIF-1α level in keloids than in normal skin tissues [21,22,44]. Moreover, Seifert *et al*. [45] reported a higher HIF-1α level in the center of the keloid than that in the periphery. HIF-1α was also shown to participate in the development of keloids by activating the TGF-β and NF-κB pathways [22]. However, the specific mechanism by which HIF-1α affects the progression of keloids is far from clear. Interestingly, a significant regulatory correlation between HOXC6 and HIF-1α expression was observed in our study. Moreover, *HIF-1α* knockdown suppressed *HOXC6* expression in KFs, suggesting that *HIF-1α* positively regulates *HOXC6* expression. Although the possible binding sites for *HIF-1α* in the *HOXC6* promoter sequence were predicted using the JASPAR database, the dual-luciferase reporter assay failed to prove that *HIF-1α* directly bound to the *HOXC6* promoter region. Epigenetic control is a proposed common mechanism of *HOX* genes. The promoter CpG islands of silenced *HOX* genes are usually methylated. The complexes of the polycomb group and trithorax induce histone trimethylation. Histone modifications change the chromatin conformation, promoting methylation or demethylation of promoter CpG islands. For instance, the *MLL* gene triggers methylation of lysine 79 on histone H3 and upregulates *HOX* genes [46]. These studies revealed that *HOX* gene expression might be altered by histone modification and methylation. A ChIP-analysis indicated that *HIF-1α* knockdown regulates histone modification of target genes under hypoxic conditions [47]. Taken together, *HIF-1α* might induce *HOXC6* expression via histone modification. Further experiments are needed to specify the molecular mechanisms underlying the interaction between *HIF-1α* and *HOXC6*, which might provide great insights into the pathogenic mechanism of hypoxia in keloids.

We conducted transcriptome sequencing analyses and *in vitro* experiments to explore the pathways downstream of HOXC6 in keloids. Transcriptome sequencing and bioinformatics analyses revealed that the ERK/MAPK-related signaling pathways were significantly enriched in the si-*HOXC6*-KF group. Our *in vitro* experiments confirmed that *HIF-1α*-induced *HOXC6* expression promoted KF proliferation, apoptosis inhibition, migration and ECM deposition through the downstream ERK pathway. As a member of the MAPK family, ERK1/2 transduces extracellular signals to intracellular signaling cascades, thereby regulating cell behaviors, including cell proliferation and differentiation [48]. Numerous studies have shown that the ERK/MAPK signaling pathway has crucial roles in tumorigenesis and metastasis [49,50]. *HOXC6* has been shown to facilitate the proliferation and migration of glioblastoma cells by activating the ERK/MAPK signaling pathway [51]. In addition, HIF-1α regulates the ERK/MAPK pathway to promote the migration and proliferation of KFs [22,52]. Overall, our results indicate the importance of the HIF-1α/HOXC6/ERK pathway in keloid growth and invasion, and further molecular mechanistic research and *in vivo* experiments are warranted to validate the clinical utility of these findings.

## Conclusions

In conclusion, our study first showed that *HOXC6* functions as an oncogenic driver in keloids by promoting KF proliferation, migration and ECM synthesis while inhibiting KF apoptosis. Furthermore, we first explored the function of the HIF-1α/HOXC6 axis via the ERK/MAPK pathway in KFs, providing candidate novel therapeutic targets for keloids. Taken together, *HOXC6* may represent a promising novel therapeutic target and a new focus for research designed to understand keloid pathogenesis.

## Authors’ contributions

KZ, QL and XP conceptualized the project and revised the manuscript. QW and YZ conducted the experiments and drafted the manuscript, contributing equally to this work. ZL and DZ collected samples and cultured the primary fibroblasts from keloid tissue samples and normal skin samples. HL, PC and CL assisted with *in vitro* experiments and data visualization. All authors read and approved the final manuscript.

**Abbreviations**
DEGs: Differentially expressed genes; ECM: Extracellular matrix; ERK: Extracellular regulated protein kinase; FBS: Fetal bovine serum; FC: Fold change; FITC: Fluorescein isothiocyanate; GAPDH: Glyceraldehyde-3-phosphate dehydrogenase; GEO: Gene Expression Omnibus; GO: gene ontology; GSEA: gene set enrichment analysis; HOXC6: homeobox C6; HIF-1α: hypoxia-inducible factor-1α; H-scores: Histoscores; KF + SCH772984: Keloid fibroblasts treated with SCH772984; KFs: keloid fibroblasts; MAPK: mitogen-activated protein kinase; Mut-pGL3-HOXC6: Mutant vector of the HOXC6 promoter; NFs: Normal skin fibroblasts; pcDNA-HIF-1α: HIF-1α overexpression vector; pcDNA-empty: Negative control vector; padj: p-Value adjusted; p-ERK: phosphorylated extracellular regulated protein kinase; PPI: Protein–protein interaction; RT-qPCR: reverse transcription-quantitative polymerase chain reaction; si-RNA: small interfering RNA; α-SMA: α*-*Smooth muscle actin; si-HOXC6-KF: Keloid fibroblasts transfected with HOXC6 siRNAs; si-NC-KF: Keloid fibroblasts transfected with the negative control siRNAs; si-NC-NF: Normal skin fibroblasts transfected with the negative control siRNAs; siRNAs: Small interfering RNAs; si-HOXC6: HOXC6 siRNAs; si-HIF-1α: HIF-1α siRNAs; si-NC: Negative control siRNAs; si-HIF-1α-KF: Keloid fibroblasts transfected with HIF-1α siRNAs; WB: Western blot; Wt-pGL3-HOXC6: HOXC6 promoter luciferase reporter vector; TGF-β1: Transforming growth factor-β1; IL: Interleukin; GSE: gene set ensemble; GSM: gene set matrix; GPL: GEO platform; DMEM: Modified Eagle’s medium; fpkm: fragments per kilobase of exon model per Million mapped fragments; AMPA: α-amino-3-hydroxy-5-methyl-4-isoxazole-propionicacid; MAP: Mitogen-activated protein; PAF: Platelet activating factor; GRB2: Growth factor receptor-bound protein-2; ERBB4: receptor protein-tyrosine kinase erbB-4; PTK6: Protein tyrosine kinase 6; SHC1: SHC transforming protein 1; mRNA: messenger RNA; ID: Identification; OD: Optical density; NS: Non-significant; HRE: HIF-1α response element.

## Supplementary Material

Supplementary_flie_tkac013

## Data Availability

The database used during the current study is available from the corresponding author upon reasonable request. The datasets (GSE145725 and GSE44270) used in this study can be found in the GEO database (https://www.ncbi.nlm.nih.gov/geo/). The original data presented in this study are included in the article. Further inquiries can be directed to the corresponding authors.
