# Supplementary material for: Multitranscriptome analyses of keloid fibroblasts reveal the role of the HIF-1α/HOXC6/ERK axis in keloid development
Source: Burns Trauma. 2022 May 9;10:tkac013. doi: 10.1093/burnst/tkac013 (PMC9085412; doi:10.1093/burnst/tkac013)
Supplement: Supplementary_flie_tkac013 [file supplementary_flie_tkac013.docx]

### Figure s1. Dual-luciferase reporter assay

### Dual-luciferase reporter assay showed that the HIF-1α overexpression vector significantly upregulated the luciferase activity of the Wt and mutant promoter sequences of HOXC6. Data are expressed as the means ± SD (n=3). **P<0.01. *HOXC6* homeobox C6, *HIF-1α* hypoxia-inducible factor-1α,

### *Wt-pGL3-HOXC6* HOXC6 promoter luciferase reporter vector, *Mut-pGL3-HOXC6* the mutant vector of the *HOXC6* promoter, *pcDNA‐HIF-1α* the *HIF-1α* overexpression vector, *pcDNA-empty* the negative control vector.
